# Aldh1 Expression and Activity Increase During Tumor Evolution in Sarcoma Cancer Stem Cell Populations

**DOI:** 10.1038/srep27878

**Published:** 2016-06-13

**Authors:** Lucia Martinez-Cruzado, Juan Tornin, Laura Santos, Aida Rodriguez, Javier García-Castro, Francisco Morís, Rene Rodriguez

**Affiliations:** 1 Hospital Universitario Central de Asturias and Instituto Universitario de Oncología del Principado de Asturias, Oviedo, Spain; 2 Unidad de Biotecnología Celular, Instituto de Salud Carlos III, Majadahonda, Madrid, Spain; 3 EntreChem SL, Oviedo, Spain

## Abstract

Tumors evolve from initial tumorigenic events into increasingly aggressive behaviors in a process usually driven by subpopulations of cancer stem cells (CSCs). Mesenchymal stromal/stem cells (MSCs) may act as the cell-of-origin for sarcomas, and CSCs that present MSC features have been identified in sarcomas due to their ability to grow as self-renewed floating spheres (tumorspheres). Accordingly, we previously developed sarcoma models using human MSCs transformed with relevant oncogenic events. To study the evolution/emergence of CSC subpopulations during tumor progression, we compared the tumorigenic properties of bulk adherent cultures and tumorsphere-forming subpopulations both in the sarcoma cell-of-origin models (transformed MSCs) and in their corresponding tumor xenograft-derived cells. Tumor formation assays showed that the tumorsphere cultures from xenograft-derived cells, but not from the cell-of-origin models, were enriched in CSCs, providing evidence of the emergence of *bona fide* CSCs subpopulations during tumor progression. Relevant CSC-related factors, such as ALDH1 and SOX2, were increasingly upregulated in CSCs during tumor progression, and importantly, the increased levels and activity of ALDH1 in these subpopulations were associated with enhanced tumorigenicity. In addition to being a CSC marker, our findings indicate that ALDH1 could also be useful for tracking the malignant potential of CSC subpopulations during sarcoma evolution.

Tumors initiate from a permissible cell-of-origin that receives the first oncogenic events needed to trigger tumoral proliferation^1^,^2^. According to the hierarchical model of cancer, after this initial step, tumors gain complexity and cellular heterogeneity, among other factors, through the emergence of tumor-propagating subpopulations or CSCs, which exhibit stem cells properties and are responsible for sustaining tumorigenesis^3^,^4^. Therefore, the evolution of these subpopulations through gaining new genetic and/or epigenetic alterations drives the evolution of tumors toward enhanced aggressiveness^5^.

Sarcomas comprise a heterogeneous group of aggressive mesenchymal malignancies that often show a limited clinical response to current therapies^6^. Experimental evidence supports the notion that many types of sarcomas are hierarchically organized and sustained by subpopulations of self-renewing CSCs that can generate the full repertoire of tumor cells and display tumor re-initiating properties^7^,^8^. In addition, it has been recently established that transformed MSCs and/or their immediate lineage progenitors are the most likely cell-of-origin for many types of sarcomas^8^,^9^,^10^. Accordingly, many of the CSC sub-populations identified in different types of sarcomas displayed MSC phenotype and functional properties^7^,^8^,^11^,^12^,^13^. Therefore, many efforts have been made to produce models of sarcomas based on MSCs transformed with relevant oncogenic events^8^,^10^. These types of models represent unparalleled systems for unraveling the mechanisms underlying sarcomagenesis from the cell-of-origin, exploring the evolution of CSC subpopulations and designing specific therapies that are able to target the tumor populations that initiate, sustain and expand the tumor.

Several methods have been developed to isolate subpopulations with stem cell properties within tumors^14^,^15^. Among these methods, the ability of certain cell subsets to grow as self-renewing tumorspheres under nonadherent and serum-starved culture conditions (sphere-formation assay) were first used to identify tissue stem cells^16^ and later CSCs from many type of tumors including sarcomas^7^,^14^,^17^,^18^,^19^. In addition, members of the aldehyde dehydrogenase family (*ALDH*) are upregulated in multipotent cells and have been proposed as potential universal markers for the identification of stem cells and CSCs from multiple sources^20^,^21^. ALDH is an enzyme responsible for oxidizing endogenous and exogenous aldehydes into their corresponding carboxylic acids^22^. Accordingly, this family of enzymes plays a self-protective, detoxifying role and therefore seems to influence the chemotherapy resistance observed in CSC subpopulations^23^. Other important functional roles of ALDH family members include participation in the retinoid signaling pathway and regulation of the expansion and differentiation of SC and CSCs^21^. Importantly, high expression and activity of members of the ALDH1 subgroup has been associated with poor prognosis and metastatic potential in several types of cancer^21^,^24^,^25^. In order to effectively use ALDH1 as a CSC marker, a method known as ALDEFLUOR assay was developed to sort cells based on their ALDH1 activity^26^. Consequently, the ALDEFLUOR assay has been widely used to isolate CSC subpopulations in various tumors including different types of sarcomas^23^,^27^,^28^,^29^,^30^,^31^. These CSC subpopulations commonly overexpress pluripotency factors such as *Sex-determining region Y-Box2* (*SOX2*), which plays important roles in the regulation of self-renewal and tumorigenicity in CSC subpopulations of several types of cancer^32^,^33^,^34^,^35^,^36^,^37^.

The process of emergence/evolution of CSC subpopulations from the cell-of-origin in sarcomagenesis has barely been addressed. In this report, we used our previously developed cell-of-origin-based models of sarcoma (Table S1)^38^,^39^,^40^ to analyze the evolution of CSC subpopulations during tumor progression. We found that the increase in the malignant potential of tumorsphere-forming subpopulations is associated with enhanced expression of SOX2 and the ALDH1 isoforms ALDH1A1 and ALDH1A3. Furthermore, ALDH1 activity correlates with tumorigenic potential during tumor progression, suggesting that this CSC marker could serve to monitor the malignant potential of CSC subpopulations during the course of the disease.

## Results

### Self-renew and differentiation properties of transformed hMSCs and their derived tumor cell lines

We have previously developed and characterized sarcoma models using human bone marrow MSCs (BM-MSCs) sequentially mutated with up to 6 oncogenic events [(1) hTERT overexpression; (2 & 3) P53 and Rb inactivation using E6 and E7 antigens of the HPV-16; (4) inactivation of PPA2 phosphatase with SV40 small T antigen (4 hits combination; MSC-4H); (5) expression of oncogenic H-RAS^v−12^ (5 hits combination; MSC-5H); and (6) the expression of FUS-CHOP (FC)] (Table S1)^38^,^39^,^40^. Three of the resulting cell lines were fully transformed and able to initiate sarcomas *in vivo*. Thus, GFP-expressing MSC-5H (MSC-5H-GFP) cells give rise to spindle cell sarcomas (SCS); meanwhile, FUS-CHOP-expressing hMSCs (MSC-4H-FC and MSC-5H-FC) originate myxoid/round cell liposarcomas (MRCLS).

**Figure 1:**
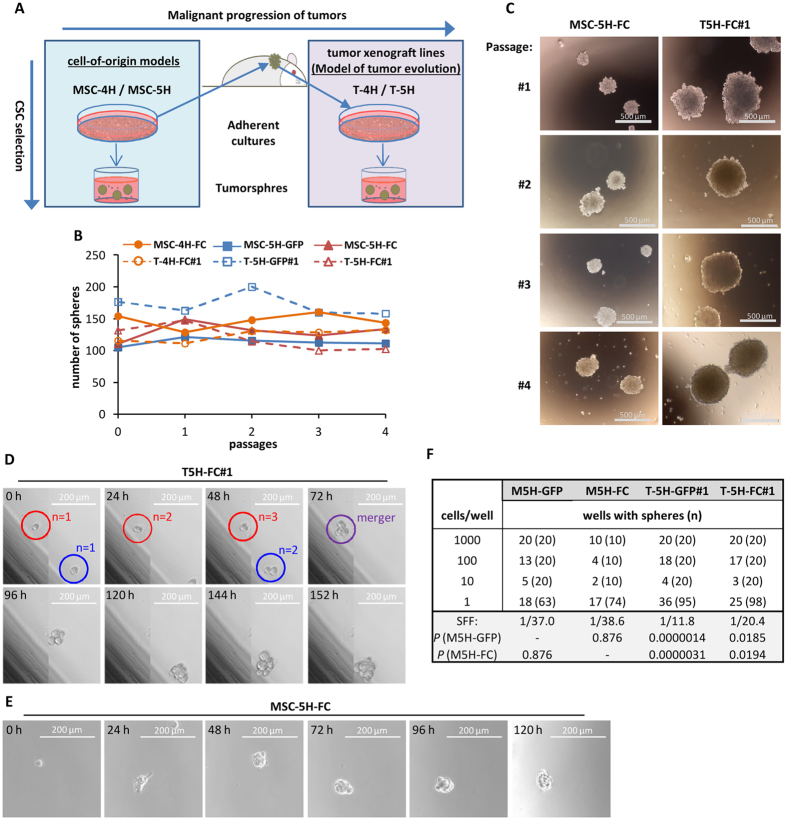
MSC-XH and T-XH cells can be serially expanded as self-renewing spheres. (**A**) The evolution of CSC subpopulations was studied by comparing the tumorsphere-forming subpopulations derived from MSC-XH cells (cell-of-origin models) *versus* those derived from their corresponding tumor xenograft-derived T-XH cells, which represent a model of malignant tumor progression. (**B–C**) Serial tumorsphere formation ability of MSC-XH and T-XH cells. Number (**B**) and representative images (**C**) of tumorspheres formed in each passage. (**D–E**) Monitoring of the the sphere formation process in T-5H-FC#1 (**D**) and MSC-5H-FC (**E**) cells by time-lapse microscopy (see also Figures S1 and S2 and Videos S1, S2 and S3). Each image is in panel D composed by two adjacent pictures automatically taken and merged by the imaging system. (**E**) Limiting dilution assay of the tumorsphere formation ability of the indicated cell lines. The number of wells presenting tumorspheres and total number of wells assayed in each condition is indicated (n). SFF was calculated using ELDA software, Pr (>chiSq) values referring to MSC-XH cells are indicated.

We first study how sphere-formation ability evolves during tumor evolution by comparing data obtained from these cell-of-origin models with data obtained from several xenograft-derived cell lines that are able to recapitulate the originally formed sarcoma (T-4H-FC#1, T-5H-GFP#1 and T-5H-FC#1)^38^,^40^ (Fig. 1A; Table S1). All cell-of-origin models [MSC-4H and MSC-5H cell types (MSC-XH)] and tumor cell lines [T-4H and T-5H cell types (T-XH)] can be serially expanded as self-renewed spheres with similar efficiency (Fig. 1B), although T-XH-derived spheres were much larger than those formed by MSC-XH cells (Fig. 1C and Figure S1). By performing time-lapse microscopy, we could monitor the sphere-formation process. Interestingly, we observed clonal division of T-5H-FC#1 cells combined with aggregation of forming spheres into bigger clusters (Fig. 1D; Figure S2; Video S1; Video S2). Similarly, in MSC-5H-FC cells, we observed both clonal tumorsphere formation (Fig. 1E; Figure S3A; Video S3) and aggregation in the initial steps of the tumorsphere-formation process (Figure S3B; Video S3). These data indicate that aggregation occurs in low density multi-cell sphere cultures even when media is supplemented with methylcellulose to reduce the mobility of the cells. In any case, a high percentage of spheres were initiated by the clonal division of a single cell and not by the aggregation of two or more cells (Video S1; Video S2; Video S3).

To further confirm the existence of cells that are able to form clonal spheres in these sarcoma models and to estimate their frequency, we performed limiting dilution assays (LDA) to detect tumorsphere formation from 1000, 100, 10 and 1 cell (Fig. 1E). Single-cell assays showed that a high percentage of cells (between 23.0% and 37.9%) were indeed able to initiate clonal growth. Sphere-forming frequency (SFF) calculated using ELDA software was also notably high in all cell types.

CSC subpopulations isolated from sarcomas have been reported to exhibit differentiation potential to MSC related lineages^7^,^12^,^41^. We previously found that this collection of sequentially mutated MSCs lost their adipogenic potential during the transformation process, and MSC-4H, MSC-5H, T-5H and T-4H cells (regardless the expression of FUS-CHOP) displayed an impaired pattern of differentiation in which most cells of the culture presented a small amount of lipid droplets in their cytoplasm. In addition, MSC-4H and MSC-5H cells retained their full ability to differentiate toward the osteogenic lineage^40^. We found that tumorspheres derived from all cell types display high osteogenic and low adipogenic potential similar to that observed in the corresponding bulk adherent cultures (Figure S4). Given that the blockage of the adipogenic differentiation pathways is a hallmark of liposarcoma development^42^, this finding is in line with a liposarcoma-forming potential of these cells when combined with other liposarcoma instructive mutations such as the expression of FUS-CHOP.

### Tumorsphere cultures from tumor-derived cells but not from the cells of origin are highly enriched in CSCs

**Figure 2:**
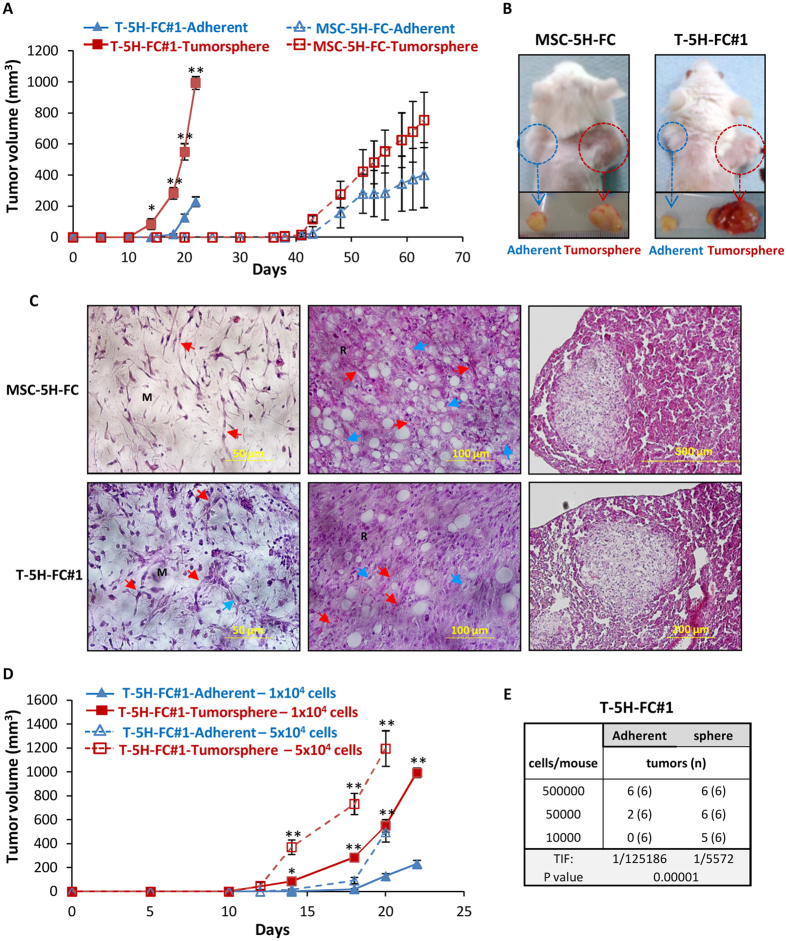
Tumor formation ability of tumorsphere cultures from MSC-5H-FC and T-5H-FC#1 cells. (**A**) Tumor growth kinetics observed after the inoculation into immunodeficient mice of 1 × 10^4^ cells from adherent or tumorsphere cultures of MSC-5H-FC and T5H-FC#1 cells. (**B**) Representative images of tumors at the experimental endpoint. (**C**) Tumor formed by both MSC-5H-FC and T5H-FC#1 cultures form tumors (left and middle panels) and lung metastasis (right panels) displaying the main characteristics of human MRCLS: myxoid matrix (M), round-cell areas (R), lipoblasts (blue arrows) and plexiform vascular pattern (red arrows). (**D**) Tumor growth kinetics observed after the inoculation into immunodeficient mice of the indicated number of cells of adherent or tumorsphere cultures of T5H-FC#1 cells. (**E**) Limiting dilution assay to evaluate tumor re-initiation ability of adherent and tumorsphere cultures of T-5H-FC#1 cells. The number of mice that grew tumors at day 14 and total number of inoculated mice for each condition is indicated. TIF was calculated using ELDA software. Error bars represent the standard deviation and asterisks indicate a statistically significant difference between the tumorspheres and adherent cell conditions (*p < 0.05, **p < 0.001; two-sided Student’s *t*-test). In limiting dilutions assays Pr (>chiSq) value is indicated.

Transplantation assays into immunocompromised mice is the current “gold standard” for identifying CSCs^1^. To check whether tumorsphere cultures of both a cell-of-origin model and their derived cell lines are enriched in tumor-initiating cells able to reproduce the original tumor, we inoculated cells from both adherent and tumorsphere cultures into NOD-SCID mice. We found that the sphere-forming subpopulation of T5H-FC#1 cells more efficiently induces tumor formation in immunodeficient mice than the bulk adherent cultures. Thus, significant differences in tumor growth were observed as early as 12 days after cell inoculation, and a 5-fold difference in tumor volume was evidenced at day 22 (Fig. 2A,B). Otherwise, MSC-5H-FC-formed spheres need a much larger latency period to induce tumor formation, and adherent cultures showed only a slight and nonsignificant delay in tumor formation when compared with the corresponding tumorsphere cultures (Fig. 2A,B). Importantly, tumorsphere cultures of both MSC-5H-FC and T-5H-FC#1 cells were able to reproduce all the features of the human MRCLS (Fig. 2C), confirming that i) MSC-5H-FC cells act as cell-of-origin for this type of tumor as we previously shown^40^, and ii) T-5H-FC#1 cultures contain CSCs that are able to reproduce all the heterogeneity of the original tumor.

To confirm and quantify the enrichment of CSCs in the tumorsphere cultures of T5H-FC#1 cells, we performed LDA assays comparing adherent and tumorsphere cultures. As shown above, tumorsphere cultures produce a significantly faster tumor growth than adherent cultures at all the assayed cell dilutions (Fig. 2D). Given that adherent cultures also contain CSCs, tumor formation were eventually observed in all cases. Therefore, we used data from the day when the tumorsphere and adherent series start to show significant differences (day 14) to perform ELDA analysis to identify CSCs in adherent and tumorsphere cultures. On this day, the inoculation of 5 × 10^5^ cells initiated tumor formation in all cases; the inoculation of 5 × 10^4^ cells produced tumors in all mice inoculated with tumorspheres, but only 2 out of 6 mice inoculated with adherent culture cells; and the inoculation of 1 × 10^4^ cells generated tumors in 5 out of 6 mice inoculated with of tumorspheres and no tumor formation was detected in the case of adherent cultures. Therefore, an ELDA analysis showed a 22-fold enrichment in tumor-initiating frequency (TIF) in tumorsphere cultures (1 in 5572) versus adherent cultures (1 in 125186) (Fig. 2E). These experiments suggest that tumorsphere cultures from the T-5H-FC#1 sarcoma-derived cell line are highly enriched in CSCs. On the other hand, their cell-of-origin cell line (MSC-5H-FC), although able to grow as floating spheres (most likely due to their intrinsic properties and not only by their tumorigenic status), could not be significantly enriched in subpopulations with increased tumorigenicity by tumorsphere culture.

### Different regulation of CSC-related factors in tumorsphere cultures of cells of origin and tumor-derived cells

**Figure 3:**
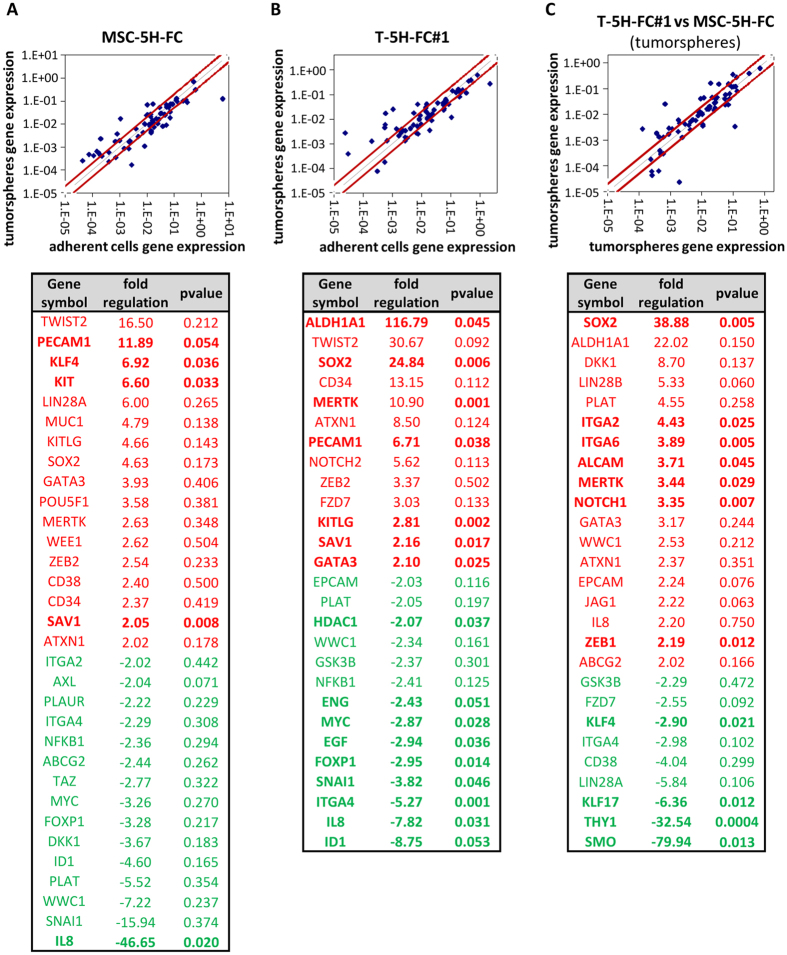
Different regulation of CSC-related factors in tumorsphere cultures of MSC-5H-FC and T-5H-FC#1 cells. RNA obtained from adherent and tumorspheres cultures of the indicated cell lines were used to analyzed the expression of 86 CSC-related genes (RT^2^ Profiler™ PCR Array System PAHS-176Z, Qiagen). (**A–C**) Upper panels show the scatter Plots representing the expression level of each gene in MSC-5H-FC spheres versus MSC-5H-FC adherent cultures (**A**), T5H-FC#1 spheres versus T-5H-FC#1 adherent cultures (**B**) and T5H-FC#1 spheres versus MSC-5H-FC spheres (**C**). Genes above and below the dark-red lines are expressed more than two fold up or down in test versus control samples. Bottom panels display the list of genes with fold change ≤−2 or ≥ 2 for each comparison. Genes showing statistically significant differences (p-value ≤ 0.05, two sided Student’s *t*-test) are depicted in bold.

To gain insight about the different abilities of tumorsphere cultures from the cell-of-origin model and the sarcoma-derived cell lines to enrich tumor re-initiating subpopulations, we performed RT-PCR in MSC-5H-FC and T-5H-FC#1 cells to analyze the expression of an array of 84 genes with well-known functions in pluripotency, self-renewal, migration, metastasis and signal transduction in CSCs as well as CSC markers. When compared to adherent cultures, tumorsphere cultures of MSC-5H-FC and T-5H-FC#1 cells showed altered expression (fold change ≥ 2) of 32 and 27 genes, respectively, although many more changes reach statistical significance (p < 0.05) in T-5H-FC#1 (16) than in MSC-5H-FC (5) (Fig. 3A,B). Differently upregulated genes in MSC-5H-FC cells include several pluripotency factors such as *KLF4*, *LIN28A* or *POU5F1/OCT4*, and CSC markers such as *KIT* and *MUC1*. Meanwhile, T-5H-FC#1 displayed a remarkable upregulation of two well-known CSC-promoting factors such as *ALDH1A1* and *SOX2*^20^,^34^,^35^ (Fig. 3A,B). Consistently, *ALDH1A1* (fold regulation: 22.02) and *SOX2* (38.88) were expressed in T-5H-FC#1-derived tumorspheres at higher levels than in those formed by MSC-5H-FC cells (Fig. 3C). Other CSC markers and signaling molecules that were upregulated in T-5H-FC#1 tumorspheres included *Dickkopf-1* (*DKK1*), *LIN28A*, *ITGA2*, *ITGA6*, *ALCAM*, *MERTK* and *NOTCH1*. Meanwhile, *SMO*, *THY1/CD90* and *KLF17* were downregulated (Fig. 3C).

### Increased expression of SOX2 and ALDH1 in CSC subpopulations during tumor progression

**Figure 4:**
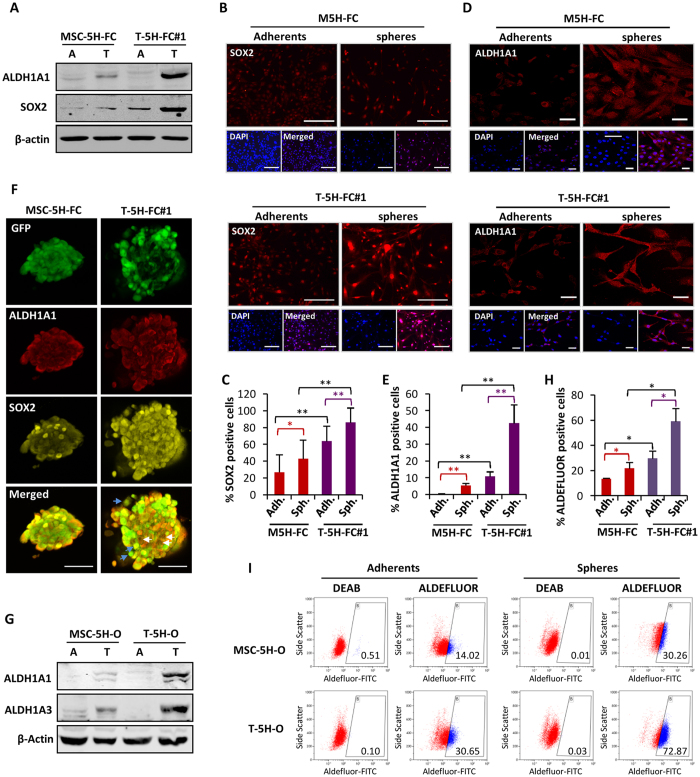
Increased expression of SOX2 and ALDH1 in CSC subpopulations during tumor progression. (**A**) Protein levels of ALDH1A1 and SOX2 in adherent (**A**) and tumorsphere (T) cultures of MSC-5H-FC and T5H-FC#1. β-actin levels were used as loading control. (**B,D**) Immunofluorescence staining of SOX2 (**B**) and ALDH1A1 (**D**) in adherent cultures or tumorsphere disaggregates and allowed to attach to glass slides of MSC-5H-FC and T5H-FC#1 cells. Scale bars = 50 μm (**B**) or = 40 μm (**D**). (**C,E**) Quantification of nuclear SOX2 (**C**) or cytosolic and nuclear ALDH1A1 (**E**) staining in each sample. (**F**) Simultaneous immunofluorescence staining of SOX2 and ALDH1A1 in GFP-expressing MSC-5H-FC- and T5H-FC#1-derived tumorspheres. Scale bars = 200 μm. (**G**) Protein levels of ALDH1A1, ALDH1A3 and β-actin in adherent (**A**) and tumorsphere (T) cultures of the indicated cell types. (**H,I**) ALDEFLUOR assay showing the activity of ALDH1 in adherents and tumorspheres cultures of MSC-5H and T5H-O cells. ALDH1 activity was blocked with the specific inhibitor DEAB to establish the basal level. The summary of 3 independent experiments (**I**) and a representative assay (**H**) is presented. Error bars represent the standard deviation and asterisks indicate a statistically significant difference between the indicated conditions (*p < 0.05, **p < 0.001; two-sided Student’s *t*-test).

According to gene expression experiments, a western blotting analysis confirmed that SOX2 and ALDH1A1 protein levels were upregulated in T-5H-FC#1 tumorspheres at a greater level than in MSC-5H-FC tumorspheres (Fig. 4A). Moreover, the immunofluorescence analysis of adherent cultures and tumorspheres that were allowed to attach to the substrate before fixation showed that T-5H-FC#1 adherent and tumorsphere cultures presented a significantly higher percentage of SOX2-positive stained nuclei than the respective nuclei of MSC-5H-FC cultures, with the nuclei of T-5H-FC#1 tumorspheres displaying the higher levels (Fig. 4B,C). Similar results were obtained after an immunofluorescence analysis of ALDH1A1 expression (cytosolic + nuclear) (Fig. 4D,E). Furthermore, *in situ* simultaneous immunofluorescent staining of SOX2 and ALDHA1 confirmed that T-5H-FC#1 tumorspheres displayed a higher proportion of cells presenting nuclear expression of SOX2 and a higher expression of cytosolic ALDH1A1 compared with MSC-5H-FC tumorspheres (Fig. 4F). Nevertheless, both populations are not totally overlapping, and there are subsets of cells expressing high levels of nuclear SOX2 but not high ALDH1A1 (Fig. 4F, blue arrows) and vice versa (white arrows).

To correlate the increased expression of ALDH1 with its enzymatic activity, we performed an ALDEFLUOR assay with adherent and tumorsphere cultures. Because this assay is based on the generation of a green fluorescent compound, we used the original MSC-5H cells (MSC-5H-O) and a cell line derived from a MSC-5H-O-generated xenograft (T-5H-O), neither transduced with GFP-expressing lentiviral vectors. We first checked the protein levels of ALDH1A1 and ALDH1A3, another ALDH1 isoform that has been reported to contribute to ALDEFLUOR activity^43^. These two isoforms were upregulated at greater levels in T-5H-O tumorspheres than in MSC-5H-O tumorspheres (Fig. 4G), as was seen when comparing MSC-5H-FC and T-5H-FC#1 cells. Similarly, the ALDEFLUOR assay showed that T-5H-O cells displayed higher levels of activity than MSC-5H-O cells in both adherent and especially tumorsphere cultures (Fig. 4H,I).

**Figure 5:**
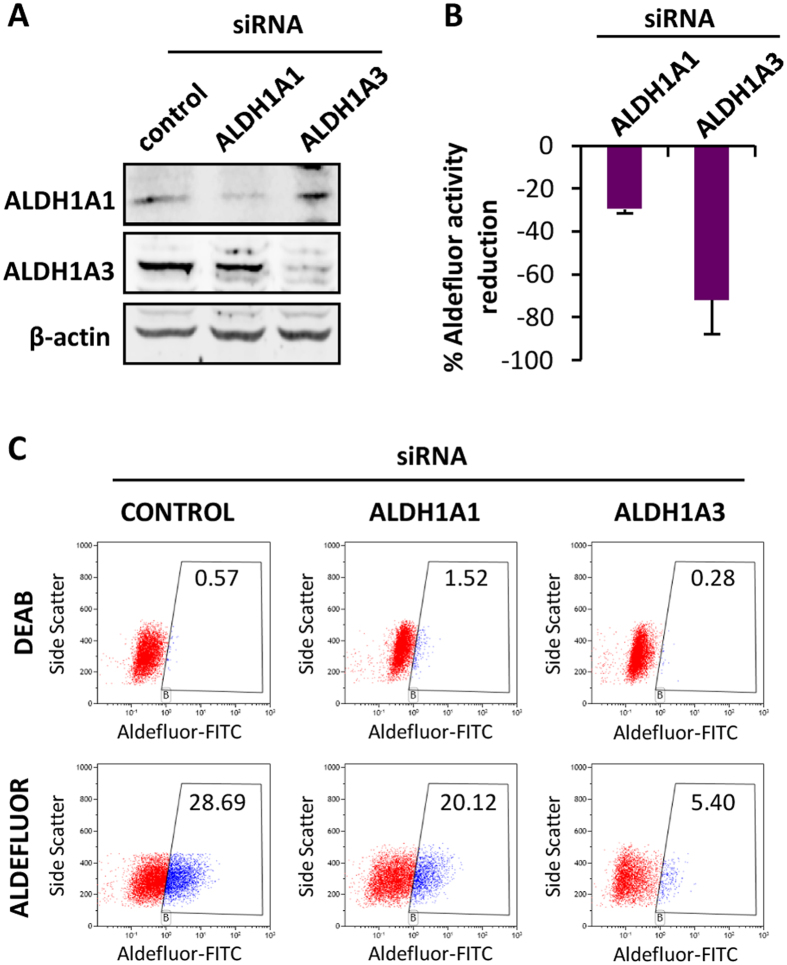
Depletion of ALDH1 isoforms result in reduced ALDEFLUOR activity. (**A**) Protein levels of ALDH1A1 and ALDH1A3 48 h after the transfection of the indicated siRNAs in T-5H-O cells. β-Actin levels are presented as a loading control. (**B,C**) ALDEFLUOR assay of T-5H-O cells depleted for ALDH1A1 or ALDH1A3. The percentage of reduction achieved by each ALDH1 isoform depletion respect to the control siRNA values (n = 3 independent experiments) (**B**) and a representative assay (**C**) is presented.

To study the contributions of ALDH1A1 and ALDH1A3 to the ALDEFLUOR activity, we performed siRNA knockdown of both isoforms in T-5H-O cells. Western blotting confirmed similar knockdown efficiency of both proteins (Fig. 5A). ALDEFLUOR assays showed that ALDH1A1 and ALDH1A3 depletion inhibited the activity by 30% and 70%, respectively, indicating that both isoforms contribute to the ALDEFLUOR activity, although ALDH1A3 seems to be the largest contributor (Fig. 5B,C).

Altogether, these results show that SOX2, ALDH1A1 and ALDH1A3 expression, together with their associated ALDEFLUOR activities are progressively enhanced in CSC subpopulations during sarcoma progression toward more aggressive phenotypes.

### ALDH1^high^ cells displayed increased tumorigenic properties

**Figure 6:**
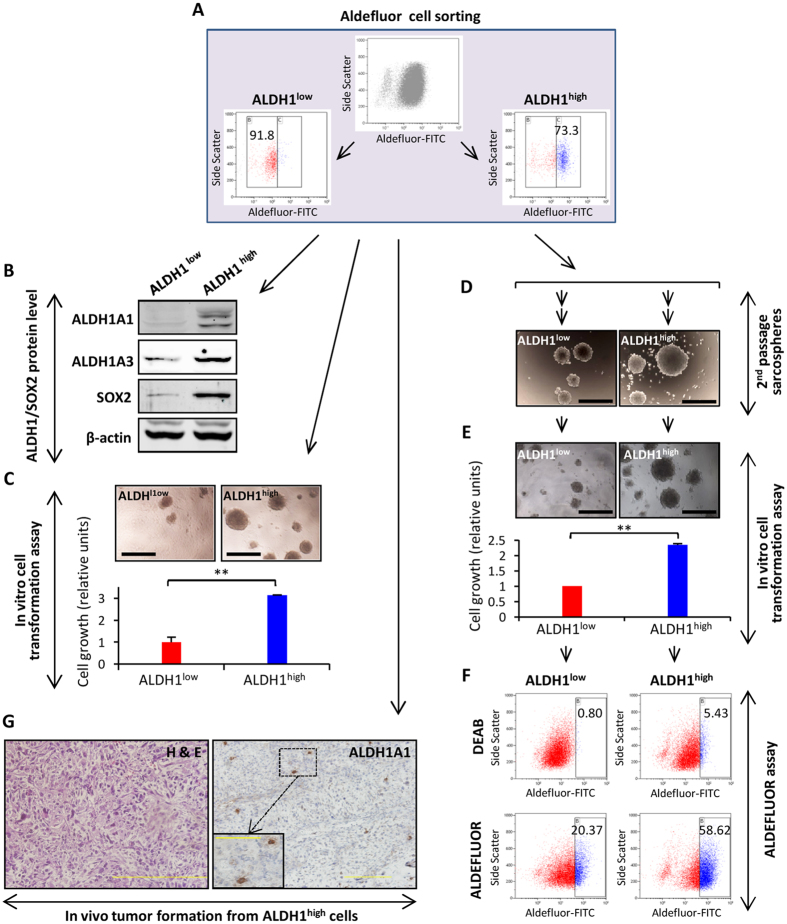
ALDH1^high^ cells displayed increased tumorogenic properties. (**A**) Flow cytometry cell sorting of ALDEFLUOR high (ALDH1^high^) and low (ALDH1^low^) populations in T-5H-O cells. (**B**) Protein levels of the indicated factors in sorted populations. β-actin levels were used as loading control. (**C**) Relative quantification of the soft agar colony formation (anchorage independent growth) ability of ALDH1^high^ and ALDH1^low^ populations. Representative images of colonies are shown (top panels). (**D–F**) Sorted cells were growth as tumorspheres for two successive passages (**D**), disaggregated and assayed for soft agar colony formation (**E**) and finally, recovered from soft agar cultures and assayed for ALDEFLUOR activity (**F**). (**G**) H&E (left panel) and ALDH1A1 immunostaining (right panel) of tumors arising in mice inoculated with 400 ALDH1^high^ cells. Scale bars = 500 μm in panels (**C–E**) and = 200 μm (100 μm for right panel inset) in panel G. Error bars represent the standard deviation and asterisks indicate a statistically significant difference between the indicated conditions (*p < 0.05, **p < 0.001; two-sided Student’s *t*-test).

To determine whether the cells presenting high ALDH1 activity are also enriched with tumor-propagating properties, we used the ALDEFLUOR assay to isolate ALDH1^high^ and ALDH1^low^ by flow cytometry (Fig. 6A). As expected, the ALDH1^high^ fraction of T-5H-O cells retained most of the expression of ALDH1A1 and ALDH1A3 proteins. Likewise, SOX2 expression was highly increased in the ALDH1^high^ fraction (Fig. 6B). Similar results were observed after the analysis of ALDH1^high^ and ALDH1^low^ populations in MSC-5H-O cells (Figure S5A). On the other hand, KLF4, which was upregulated in tumorsphere cultures of MSC-5H-FC cells, was not enriched in ALDH^high^ sorted populations of MSC-5H-O or T-5H-O cells (Figure S5B), similar to previous observations in osteosarcoma samples^44^.

Notably, the ALDH1^high^ fraction in T-5H-O cells presented a higher ability to form colonies in soft agar, a surrogate *in vitro* transformation assay (Fig. 6C). In contrast, ALDH1^high^ and ALDH1^low^ populations showed similar abilities to form serially passaged tumorspheres (results from two separate experiments shown in Fig. 6D and Figure S6A). To further analyze the self-renewal and tumorigenic properties of both populations, second-passage tumorsphere cultures from ALDH1^high^ and ALDH1^low^ cells were disaggregated and assayed form colony formation in soft agar. Again, ALDH1^high^ cells displayed enhanced cell growth in these conditions (Fig. 6E and Figure S6B). Moreover, the ALDEFLUOR assay performed with the cells recovered from the *in vitro* transformation assay reveled that ALDH1^high^ cells maintain high levels of ALDH1 activity while the ALDH1^low^ cells were moderately enriched in ALDH1 activity after tumorsphere culture (Fig. 6F and Figure S6C).

Finally, we found that as few as 400 ALDH1^high^ cells were sufficient to develop tumors *in vivo* with 100% of incidence (n = 6). The immunohistological analysis showed that these tumors resembled the original SCS histology and that only a small portion of tumor cells (<5%) expressed high levels of ALDH1A1 (Fig. 6G). These results indicate that high activity of ALDH1 is strongly related to increased tumorigenic properties and that ALDH1^high^ cells behave as true CSC subpopulations able to regenerate all the tumor cell subpopulations.

## Discussion

To gain insights about the evolution of CSC subpopulations during tumor progression in sarcomas, we compared the tumorsphere-forming subpopulations derived from MSC-XH cells (cell-of-origin models) vs. those derived from their corresponding tumor xenograft-derived T-XH cells (Fig. 1A)^38^,^40^. We observed that both MSC-XH and T-XH cells formed clonal tumorspheres with very high efficiency. In this regard, it is likely that the intrinsic self-renewal properties of the MSCs^45^,^46^ acting as cell-of-origin for sarcomas could result in higher frequencies of tumorsphere formation upon tumoral transformation. In fact, high frequencies of tumorsphere formation have previously been reported in some cases of sarcoma^12^. Notably, certain cautions have been raised about the use of tumorsphere cultures to enrich CSC subpopulations in some cases. Thus, using imaging approaches, neurospheres were observed to frequently aggregate even at low densities^47^. Similarly, using time-lapse microscopy, we showed that in low-density multi-cell tumorsphere cultures, aggregation occurs even in the presence of methylcellulose. In any case, our experiments also demonstrate that clonal proliferation takes place before and after sphere aggregation both in MSC-5H-FC and T-5H-FC#1 cells. Importantly, we found remarkable differences in the ability of these clonal/aggregated tumorsphere cultures from these two cell types to effectively enrich in tumor-initiating populations (Table 1). On the one hand, tumorsphere cultures from the T-5H-FC#1 sarcoma-derived cell line are highly enriched in CSCs as seen by their highly increased capacity to initiate tumor formation *in vivo*. On the other hand, their cell-of-origin cell line (MSC-5H-FC), although able to grow as floating spheres, could not be significantly enriched in subpopulations with increased tumorigenicity by tumorsphere culture. Altogether, this model of sarcomagenesis evolution suggests the differences between a non-heterogeneous cell-of-origin population able to initiate sarcomagenesis (MSC-5H-FC cells) and the increasingly aggressive CSC subpopulations that emerge during *in vivo* tumor progression (T-5H-FC#1 cells). These tumor-propagating subpopulations most likely appear and evolve through the accumulation of epigenetic alterations and are a source of intra-tumor heterogeneity^1^. These results also reflect the importance of performing *in vivo* tumor-formation experiments to confirm the ability of tumorsphere cultures to enrich in bona fide CSC subpopulations.

By analyzing the expression of several genes involved in the CSC phenotype in MSC-5H-FC and T-5H-FC#1 adherent and tumorsphere cultures, we found a group of genes highly enriched in T-5H-FC#1 vs. MSC-5H-FC tumorsphere cultures that are therefore progressively increased in tumorsphere-forming subpopulations during sarcoma progression. Among these genes, *ALDH1A1* and *SOX2* were the most highly upregulated in T-5H-FC#1 tumorspheres and may be meaningful indicators of CSC progression in sarcomagenesis and also useful for the prospective isolation of CSCs. Both factors were also upregulated at protein levels and their ALDEFLUOR activities were equally enhanced in T-5H vs. MSC-5H tumorspheres (Table 1). In line with this finding, the upregulation of the mRNA levels of *ALDH1A1*^23^ and *SOX2*^12^,^18^,^19^,^23^ were already detected in tumorsphere cultures of sarcomas.

It is important to note that apart from ALDH1A1, other members of the ALDH superfamily, especially ALDH1A3, seem to contribute to ALDEFLUOR activity^43^,^48^. We confirmed that, similar to ALDH1A1, ALDH1A3 was upregulated in tumorsphere cultures. Likewise, cells with high ALDEFLUOR activity retain most of the ALDH1A1 and ALDH1A3 expression, and both isoforms contribute importantly to ALDEFLUOR activity in our models. In any case, we could not discard a potential role for other members of the ALDH family.

ALDH1 activity has been used to isolate CSC subpopulations in different types of sarcomas^23^,^27^,^28^,^29^,^30^,^31^. These studies found that subpopulations with high activity of ALDH1 showed increased expression of pluripotency markers like SOX2, enhanced ability to grow as tumorspheres, increased tumorigenicity and strong chemo-resistance. In line with these findings, we found that SOX2 expression was highly increased in ALDH1^high^ cells. However, we observed that both ALDH1^high^ and ALDH1^low^ populations are able to grow as tumorspheres, indicating that other markers or combinations of markers are needed to discriminate tumorsphere-forming populations. In any case, ALDH1 activity was always enriched in these growth conditions and ALDH1^high^ cells possess high self-renewal potential as indicated by their ability to grow as serially passaged spheres that maintain high ALDH1 activity. Importantly, we also found that ALDH1 activity in T-5H cells was associated with enhanced tumorigenic properties. Therefore, our findings correspond to previous studies suggesting that ALDH1 seems to constitute a valuable CSC marker in sarcomas. Moreover, we present for the first time evidence that ALDH1 expression and activity is increased during tumor evolution in CSC subpopulations (T-5H vs. MSC-5H tumorsphere cultures), suggesting that the level of ALDH1 could be used as an indicator of the evolution of the CSCs’ malignant potential.

Similar to ALDH1, our results show that SOX2 is upregulated in CSC subpopulations during tumor evolution. Importantly, the depletion of SOX2 has been reported to induce a significant decrease in the tumor-initiating capability of ALDH1^high^ cells in melanoma^37^. We found that SOX2 was highly enriched in ALDH^high^ populations, although certain level of SOX2 expression was detected in ALDH^low^ cells. In addition, immunofluorescence analysis of tumorspheres that are enriched in ALDEFLUOR activity showed cells expressing different levels of SOX2. Altogether, these data suggest that the selection of SOX2^+^/ALDH^high^ cells could result in a better discrimination of CSC subpopulations. As commented before, live cells with high ALDH1 activity can be easily sorted using the ALDEFLUOR assay. Unfortunately, the sorting of live cells based on the level of SOX2 expression cannot yet be achieved despite some promising technical advances^49^. Once this technology becomes available, it would be able to separate subpopulations presenting high activity or expression of both ALDH1 and SOX2 and could potentially represent tumor-propagating subpopulations better than the cell subset solely selected by ALDH1 activity.

Beside *ALDH1A1* and *SOX2*, other molecules such as *DKK1* and *NOTCH1* were notably upregulated in CSCs during tumor progression. Notably, the WNT-antagonist DKK1 has been proposed to enhance pro-tumorigenic properties in osteosarcoma, in part through the upregulation of ALDH1A1^50^. Likewise, NOTCH signaling has been associated with ALDH activity and increased metastatic potential in osteosarcoma cells^51^.

In conclusion, our model of sarcomagenesis initiated from transformed BM-MSCs, allowing us to study the evolution of the tumor from the cell-of-origin toward increasingly aggressive xenograft-derived cells. By comparing the tumorigenic properties of the tumorsphere-forming populations derived from both populations, we evidenced the emergence of *bona fide* CSC subpopulations during tumor progression. Several factors related with the CSC phenotype, such as *ALDH1A1*, *SOX2*, *DKK1* and *NOTCH1*, were increasingly upregulated in the emerging CSC subpopulations during tumor progression. Importantly, the increased levels of ALDH1 expression and activity in these subpopulations was associated with enhanced tumorigenicity, thus confirming the suitability of this molecule to be used as a CSC marker for sarcomas and also suggesting that ALDH1 could be useful for tracking the malignant potential of CSC subpopulations during tumor evolution and treatment.

## Methods

### Cell types

Human BM-MSCs sequentially mutated with up to 6 oncogenic events, and tumor lines derived from transformed BM-MSC-induced xenografts were previously generated and characterized (Table S1)^38^,^39^,^40^. The identity of transformed human BM-MSCs has been authenticated by a Short Tandem Repeats analysis during the last 5 months. All the cell types were cultured as previously described^40^,^52^. All experimental protocols have been performed in accordance with institutional review board guidelines and were approved by the Institutional Ethics Committee of the Hospital Universitario Central de Asturias. All samples from human origin were obtained upon signed informed consent.

### Tumorsphere culture

Cells lines were plated at a density of 5,000 cells per well in 6-well plates (2.5 cell/μl) treated with a sterile solution of poly 2-hydroxyethyl methacrylate (10 g/l in 95% ethanol; Sigma) to prevent cell attachment, in serum-free sphere medium containing DMEM-F12 (GE Healthcare, Pittsburg, PA) supplemented with Glutamax (1:100; Life Technologies, Carlsbad, CA), B-27 Supplement (1:50; Life Technologies), Heparin (1:1000; Sigma, St Louis, MO), the growth factors human EGF (20 ng/ml) and human bFGF (10 ng/ml; PeproTech, London, UK) and 1% methylcellulose (Sigma) to avoid cell aggregation. In addition, fresh aliquots of EGF and bFGF were added every three days. After 10–12 days, well-formed spheres were filtered through 70-μm cell strainers (Corning, New York, NY), washed with phosphate-buffered saline and disaggregated by incubation with trypsin (0.25%)/EDTA (Life Technologies) for 15 min. The resulting cell suspension was pelleted, counted and replated as before to continue serially passaged tumorsphere cultures. In LDA, serially diluted numbers of cells (from 1000 to 1 cell) were plated in poly 2-hydroxyethyl methacrylate-treated 96-well plates and cultured as explained above. Presented data are aggregates of two independent experiments (Supplemental Material). After 10 days, the number of wells presenting spheres were counted and the sphere-forming frequency (SFF) was calculated using the ELDA software^53^. Tumorsphere formation was monitored using a Zeiss Cell Observer Live Imaging microscope (Zeiss, Thornwood, NY) coupled with a CO2 and temperature-maintenance system. Time-lapse images were acquired every 8 hours during 6 days using a Zeiss AxioCam MRc camera.

### Analysis of CSC-related genes using RT-PCR arrays

The Human Cancer Stem Cells RT2 Profiler PCR Array (PAHS-176-Z; SABiosciences, Qiagen Iberia, Madrid, Spain) was used to analyze the expression of 84 genes linked to CSCs properties according to the manufacturer instructions. Only RNA samples with RNA integrity ≥ 9 were used in the analysis as determined using an Agilent 2100 Bioanalyzer (Agilent Technologies, Santa Clara, CA). The RT-qPCR reactions were performed for three independent experiments of each condition in a StepOnePlus Real Time PCR system (Life Technologies, Carlsbad, CA) using the RT^2^ First Strand Kit and the RT^2^ qPCR SYBR Green/ROX MasterMix (SABiosciences). The PCR conditions included an initial denaturation step at 95 °C for 10 minutes, followed by 40 cycles of a denaturation step at 95 °C for 15 seconds and an annealing/extension step at 60 °C for 1 minute. A final dissociation curve was generated to verify that a single product was amplified. Reactions in the absence of template and in the absence of enzyme were also included as negative controls. A complete data set, including gene information and experimentally obtained C_t_ values, is presented in Table S2. The RT-qPCR raw data were analyzed using the PCR Array Data analysis template (SABiosciences) and software available online at the SABiosciences website: www.sabiosciences.com/pcr/arrayanalysis.php, which provides a statistical analysis of data. Genes presenting amplification Ct values > 35 in both untreated and treated cells were discarded. Relative expression values of the different genes were calculated from the threshold cycle (Ct) following the DDCt method using *ACTB*, *B2M*, *GAPDH*, *HPRT1* and *RPLP0* as reference genes.

### Xenograft experiments

Female NOD/SCID mice of 5–6 weeks old (Janvier Labs, St Berthevin, France) were inoculated subcutaneously (s.c.) with 5 × 10^5^, 5 × 10^4^ or 1 × 10^4^ T5H-FC#1 / MSC-5H-FC cells or 400 T-5H-O cells mixed 1:1 with BD Matrigel Matrix High Concentration (BD Biosciences, Erembodegem, Belgium) previously diluted 1:1 in culture medium. Tumor size was measured with a caliper 2–3 times a week and tumor volume was determined using the equation (D × d^2^)/6 × 3.14, where D is the maximum diameter, and d is the minimum diameter. Tumor volumes for all mice in each xenograft-treatment group were averaged to obtain the mean tumor volume for the corresponding group. Student’s *t*-test was performed to determine the statistical significance between control and treated groups. Animals were sacrificed when the tumors formed from tumorsphere cultures reached approximately 1,000 mm^3^. Relative tumor-initiating frequency (TIF) was calculated using the data obtained at the day that differences in tumor volume between tumorsphere and adherent groups became statistically significant (day 14 after inoculation) using the ELDA software^53^. Upon removal, tumor samples were fixed in formol, embedded in paraffin, cut into 4-μm sections, and stained with hematoxylin and eosin (H&E) and anti-ALDH1A1 [(ab105920), 1:400 dilution] from Abcam (Cambridge, UK) as previously described^54^. All experimental protocols were carried out in accordance with the institutional guidelines of the University of Oviedo and were approved by the Animal Research Ethical Committee of the University of Oviedo prior to the study.

### Western blot

Whole cell protein extraction and western blot analysis were performed as previously described^55^. The antibodies used in Western blot analysis were as follows: anti-ALDH1A1 [(ab105920), 1:1,000 dilution] from Abcam (Cambridge, UK); anti-ALDH1A3 [(AP7847a), 1:50 dilution] from Abgent (San Diego, CA); anti-SOX2 [(PA1-094), 1:1,000 dilution] from Thermo Fisher (Waltham, MA); and anti-β-Actin [(A-1978), 1:20,000 dilution] from Sigma.

### Immunofluorescence staining

For the 2-D immunofluorescence staining experiments, adherent and tumorsphere cultures were left to attach to glass coverslips, fixed with 4% formaldehyde (Sigma) for 15 min at room temperature (RT) and permeabilized in PBS containing 0.2% triton X-100 (Sigma) for 5 min at 4 °C. Cells were then blocked with PBS containing 1% BSA and 0.05% Tween 20 (PBT) for 5 min at RT and incubated with 1:200 diluted mouse monoclonal anti-ALDH1A1 (ab105920 from Abcam,) or 1:250 diluted rabbit monoclonal anti-SOX2 (PA1-094 from Thermo Fisher) for 24 hours, washed 3 times with PBS and incubated with 1:300 anti-mouse alexa fluor 555 (A-21422 from Thermo Fisher) or 1:300 anti-rabbit alexa fluor 555 (A-21428 from Thermo Fisher) for 1 hour in the dark. Antibody dilutions and washes after incubations were conducted in PBT. Coverslips were finally mounted in Vectashield mounting medium with DAPI (Vector Laboratories Inc.; Burlingame, Ca).

In the 3-D staining experiments, tumorspheres cultures from GFP-positive cells were washed with phosphate-buffered saline (PBS) twice and fixed in 4% paraformaldehyde (Sigma) in PBS for 20 minutes. Tumorspheres were then washed 3 times with PBS, permeabilized in PBS plus 0.1% triton X-100 (Sigma) for 20 minutes, washed another 5 times with PBS and incubated with the blocking solution (10% goat serum in PBS) for 1 hour. Afterward, tumorspheres were incubated with rabbit monoclonal anti-SOX2 [1:150 dilution] and mouse monoclonal anti-ALDH1A1 [1:150 dilution] for 36 hours, washed 4 times with PBS plus 0.1% Tween-20 for 10 minutes and incubated with anti-mouse alexa fluor 555 (1:300 dilution) and anti-rabbit alexa fluor 647 IgG [1:300 dilution (ab150079 from Abcam)] for 1 hour. Finally, the slides were washed extensively with PBS, pipetted into a drop of ProLong^®^ Gold Antifade Mountant medium (P36930 from Life Technologies) and deposited in μ-Slide 8-well chambered coverslips (80826 from Ibidi; Planegg, Germany).

Confocal section and projection images collected with identical exposure times were obtained using an Ultra-Spectral TCS-SP2-AOBS confocal microscope (Leica, Wetzlar, Germany). Quantification of positive nuclei (SOX2) or nuclei + cytoplasm (ALDH1A1) in 2D experiments were performed in 10-12 randomly selected fields using Confocal Uniovi Image J software 1.5.1 (University of Oviedo, Spain; http://www.sct.uniovi.es/confocaluniovi) based in the Image J software (National Institute of Health, Bethesda, MD).

### Aldefluor assay

ALDH activity was determined using the activated Aldefluor^TM^ reagent, a fluorescent non-toxic substrate for ALDH1 able to freely diffuse into intact and viable cells (Stem Cells Technologies, Grenoble, France). 1 × 10^6^ cells were suspended in 1 ml of Aldefluor assay buffer containing the ALDH1 substrate (Bodipy-Aminoacetaldehyde) and incubated for 45 min at 37 °C. As a reference control, the cells were suspended in buffer containing the substrate in the presence of diethylaminobenzaldehyde (DEAB; 20 μM for the experiments described in Fig. 4 and 10 μM for the rest of the experiments), a specific ALDH1 enzyme inhibitor. Of note, the lower concentration of DEAB failed to fully inhibit high ALDEFLUOR activities (e.g., those observed in tumorsphere cultures of T-5H-O cells) as previously reported^48^. In any case, gates were established to include less than 1% positive cells in the DEAB controls of the condition displaying less ALDEFLUOR activity in adherent or tumorsphere cultures and were maintained in all conditions. Cells were incubated with 0.5 μg/ml propidium iodide for 15 min, and cells positive for this staining (dead cells) were excluded from the analysis (Figure S7). The brightly fluorescent ALDH1-expressing cells (ALDH1^high^) were detected and sorted using the green fluorescence channel (520–540 nm) of a MoFlo XDP flow cytometry (Beckman Coulter, Brea, CA).

### siRNA transfection

ALDH1A1 (J-008722-06-0002) and ALDH1A3 (J-009082-07-0002) On-Target plus siRNAs and siGenome RiSC-Free control siRNA (D-001220-01) were from Dharmacon (Lafayette, CO). SiRNA sequences were as follows: ALDH1A1: GAACAGUGUGGGUGAAUUG; ALDH1A3: UGAAUACGCUUUGGCCGAA. Cells were transfected with 200 pmol/ml siRNAs using Lipofectamine 3000 (Life Technologies) according to the manufacturer’s instructions. 48 hours after transfection, cells were collected and analyzed^56^.

### Soft agar colony formation assay

A soft agar colony formation assay was carried out using the CytoSelect^TM^ 96-Well Cell Transformation Assay Kit (Cell Biolabs Inc, San Francisco, CA) as described^57^. Quantification of anchorage-independent cell growth was performed after agar layer solubilization and cell recovering according to the manufacturer’s instructions. Cell viability was analyzed using the Cell Proliferation Reagent WST-1 (Roche, Mannheim, Germany). Briefly, recovered cells were plated in 24-well plates, left to attach to the plastic substrate (4–6 hours) and incubated for 1 hour at 37 °C with 18 μl/ml of WST-1 reagent. The color change produced by the cleavage of the WST-1 reagent by mitochondrial dehydrogenases was measured by reading the absorbance at 440 nm with the use of a Synergy HT plate reader (BioTek, Winooski, VT). The surviving fraction at each treatment was calculated as a percentage of untreated cells. Two independent experiments were performed in triplicate.

## Additional Information

**How to cite this article**: Martinez-Cruzado, L. *et al*. Aldh1 Expression and Activity Increase During Tumor Evolution in Sarcoma Cancer Stem Cell Populations. *Sci. Rep*. **6**, 27878; doi: 10.1038/srep27878 (2016).

## Supplementary Material

Supplementary Information

Supplementary Movie S1

Supplementary Movie S2

Supplementary Movie S3

Supplementary Data S1

## Figures and Tables

**Table 1:** Main differences observed between the cell-of-origin models and their derived tumor xenograft cell lines.

	Cell of origin model (MSC-5H)	model of tumor evolution (T-5H)
intra-tumor heterogeneity	low	increased
sphere forming ability*	+++ (small spheres)	+++ (large spheres)
selection of tumor initiating cells by tumorsphere culture*	+	+++
increase of ALDH1A1 in tumorspheres*	+	+++
increase of ALDH1A3 in tumorspheres*	+	+++
levels of ALDEFLUOR activity*	++	+++
increase of SOX2 in tumorspheres*	+	+++
increase in sphere forming ability in ALDH^high^ cells*	n.a.	−
increase of tumorigenic properties in ALDH^high^ cells*	n.a	++

(*) Relative level of increase in the indicated properties: (−): no differences; (+): small increase; (++): moderate increase; (+++): large increase; n.a.: not analyzed.
