# Supplementary material for: Aldh1 Expression and Activity Increase During Tumor Evolution in Sarcoma Cancer Stem Cell Populations
Source: Sci Rep. 2016 Jun 13;6:27878. doi: 10.1038/srep27878 (PMC4904413; doi:10.1038/srep27878)
Supplement: Supplementary Information [file srep27878-s1.pdf]

ALDH1 EXPRESSION AND ACTIVITY INCREASE DURING TUMOR EVOLUTION IN SARCOMA CANCER STEM CELL POPULATIONS

Lucia Martinez-Cruzado[1], Juan Tornin[1], Laura Santos[1], Aida Rodriguez[1], Javier García Castro[2], Francisco Morís[3], and Rene Rodriguez[1*]

[1]Hospital Universitario Central de Asturias and Instituto Universitario de Oncología del Principado de Asturias, Oviedo, Spain. [2]Unidad de Biotecnología Celular, Instituto de Salud Carlos III, Majadahonda, Madrid, Spain. [3]EntreChem SL, Oviedo, Spain.

SUPPLEMENTAL INFORMATION

METHODS

Osteogenic and adipogenic differentiation assays

hMSCs from adherent cultures or dissagregated tumorspheres were plated at $5x10^4$ cells/cm^2. At confluence, growth medium was replaced with specific differentiation inductive medium. For adipogenic differentiation, cells were cultured in Adipogenic MSCs Differentiation Bullet Kit (Lonza, Basel, Switzerland) for 2 weeks. Differentiated cell cultures were stained with Oil Red O (Sigma). For osteogenic differentiation, cells were cultured in Osteogenic MSCs Differentiation Bullet Kit (Lonza) for 2 weeks. Differentiated cultures were stained with Alizarin Red S (Sigma).

Tumorsphere-formation limiting dilution assays

Serially diluted numbers of cells (from 1000 to 1 cell) were plated in poly 2-hydroxyethyl methacrylate-treated 96 well plates and cultured in tumorsphere culture conditions. The number of wells plated for each cell dilution in each cell line was as follow:

MSC-5H-GFP: 10 wells with 1000 cells; 10 wells with 100 cells; 10 wells with 10 cells; and 66 wells with 1 cell (two experiments).

MSC-5H-FC: 5 wells with 1000 cells; 5 wells with 100 cells; 5 wells with 10 cells; and 81 wells with 1 cell (two experiments).

T-5H-GFP#1 and T-5H-FC#1: 10 wells with 1000 cells; 10 wells with 100 cells; 10 wells with 10 cells; and 96 wells with 1 cell (two experiments).

After plating of the 1-cell dilution, wells were cheked and those presenting no cells or more than one cell were discarded and not included in subsequent analysis. After 10 days, the number of wells presenting spheres were counted and the sphere forming frequency (SFF) was calculated using the ELDA software.

RT-PCR analysis

Total RNA was extracted using GeneMatrix Universal RNA Purification Kit (EURX, Gdańsk, Poland). One microgram of RNA was used for each RT reaction, which was performed using the Maxima First Strand cDNA Synthesis Kit for RT-qPCR (Thermo Fisher, Waltham, MA). The expression of KLF4 was determined by Taqman probe based qRT-PCR using Taqman universal Pcr Master Mix (Applied Biosystems, Foster City, CA). Actin was used as a housekeeping gene. The following PCR conditions were used: 40 cycles of 2 minutes at 50 ^{o}C followed by 10 minutes at 95 ^{o}C followed by 15 seconds at 95 ^{o}C and 60 seconds at 60 ^{o}C. Primer sequence references are as follows: KLF4 (Hs00358836_m1; Applied Biosystems) and actin (4352935E; Applied Biosystems).

VIDEO LEGENDS

Video S1. Sphere formation process in T-5H-FC#1 cells monitored by time-lapse microscopy. Time-lapse images were acquired every 8 hours during 6 days using a Zeiss AxioCam MRc camera.

Video S2. Sphere formation process in T-5H-FC#1 cells monitored by time-lapse microscopy. Time-lapse images were acquired every 8 hours during 6 days using a Zeiss AxioCam MRc camera.

Video S3. Sphere formation process in MSC-5H-FC cells monitored by time-lapse microscopy. Time-lapse images were acquired every 8 hours during 6 days using a Zeiss AxioCam MRc camera.

SUPPLEMENTAL TABLE

Supplementary table 1. Transforming mutations and oncogenic status of the MSC cell types.

BM-hMSC	hTERT	E6-HPV16 (inactivat. of P53)	E7-HPV16 (inactivat. of RB)	SV40 ST antigen (inactivat. of PPA2)	H-RAS^{v-12}	FUS-CHOP	GFP	Oncogenic status	Tumor type
**BMSC**	-	-	-	-	-	-	-	wild type	-
**MSC-4H-FC**	+	+	+	+	-	+	+	transformed	myxoid liposarcoma
**T-4H-FC#1**	+	+	+	+	-	+	+	xenograft-derived line	myxoid liposarcoma
**MSC-5H-O**	+	+	+	+	+	-	-	transformed	spindle cell sarcoma
**MSC-5H-GFP**	+	+	+	+	+	-	+	transformed	spindle cell sarcoma
**MSC-5H-FC**	+	+	+	+	+	+	+	transformed	myxoid liposarcoma
**T-5H-O**	+	+	+	+	+	-	-	xenograft-derived line	spindle cell sarcoma
**T-5H-GFP#1**	+	+	+	+	+	-	+	xenograft-derived line	spindle cell sarcoma
**T-5H-FC#1**	+	+	+	+	+	+	+	xenograft-derived line	myxoid liposarcoma

SUPPLEMENTAL FIGURES

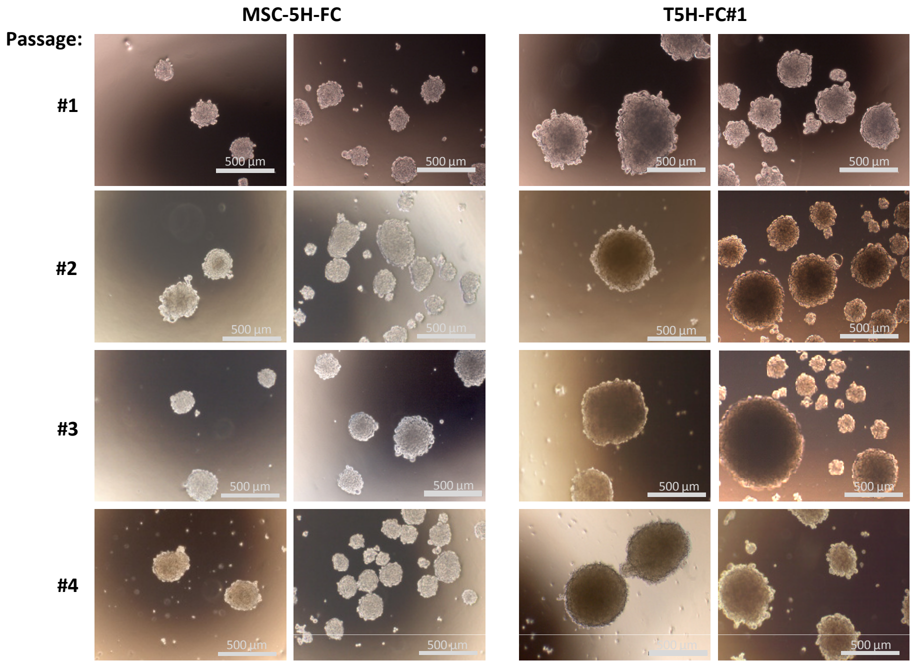

Figure S1: Tumorsphere cultures of MSC-5H-FC and T-5H-FC#1 cells lines. Representative images of seriallly passaged tumorsphere cultures.

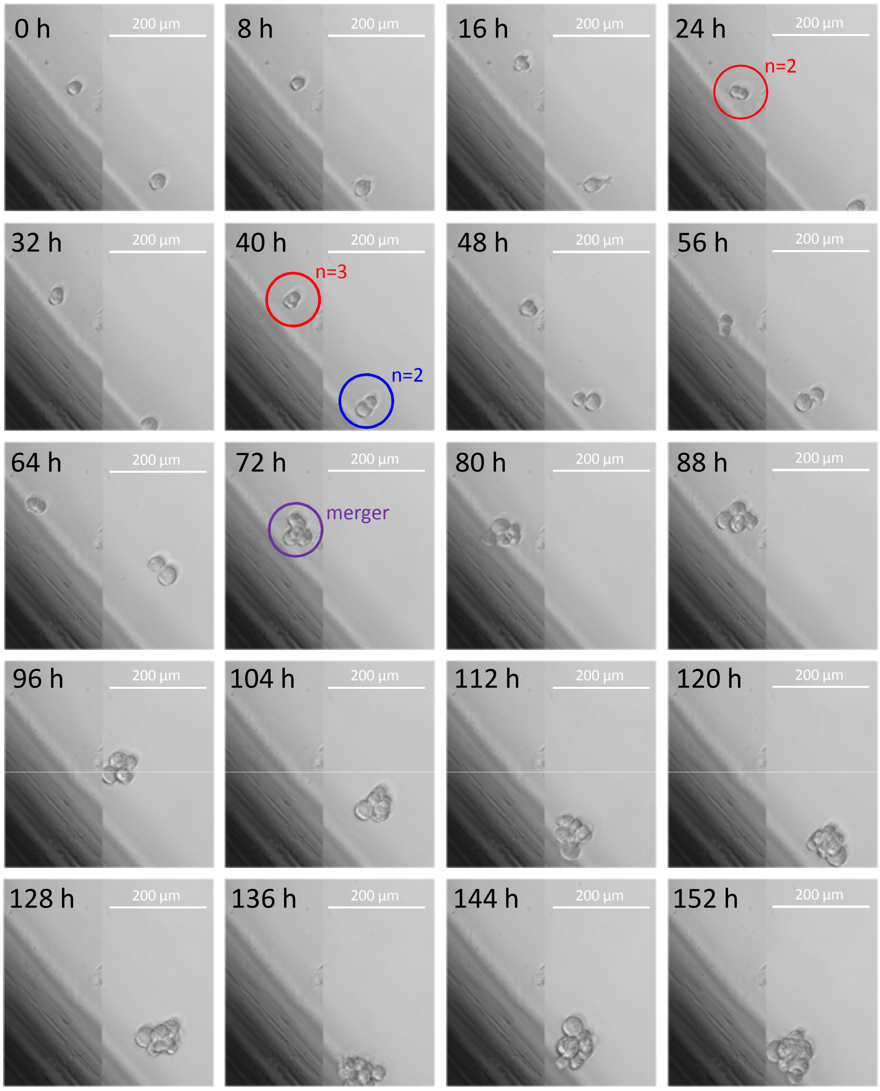

Figure S2: Sphere formation process in T-5H-FC#1 cells monitored by time-lapse microscopy. The figure shows the complete set of images of the experiment shown in figure 1D.

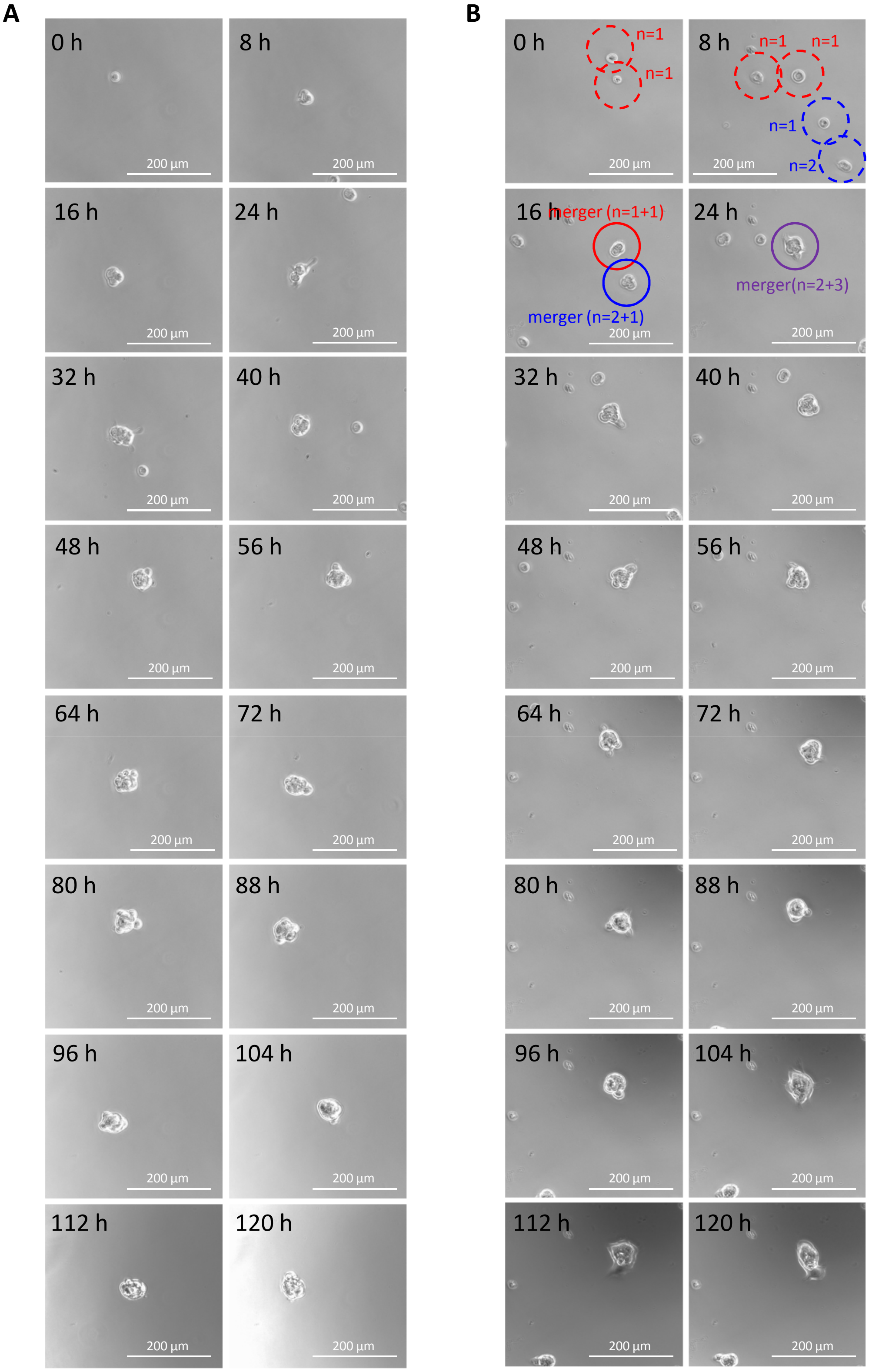

Figure S3: Sphere formation process in MSC-5H-FC cells monitored by time-lapse microscopy. (A) Tumorsphere formed by clonal growth (complete set of images of the experiment shown in figure 1E). (B) Tumorsphere formed after initial aggregation steps.

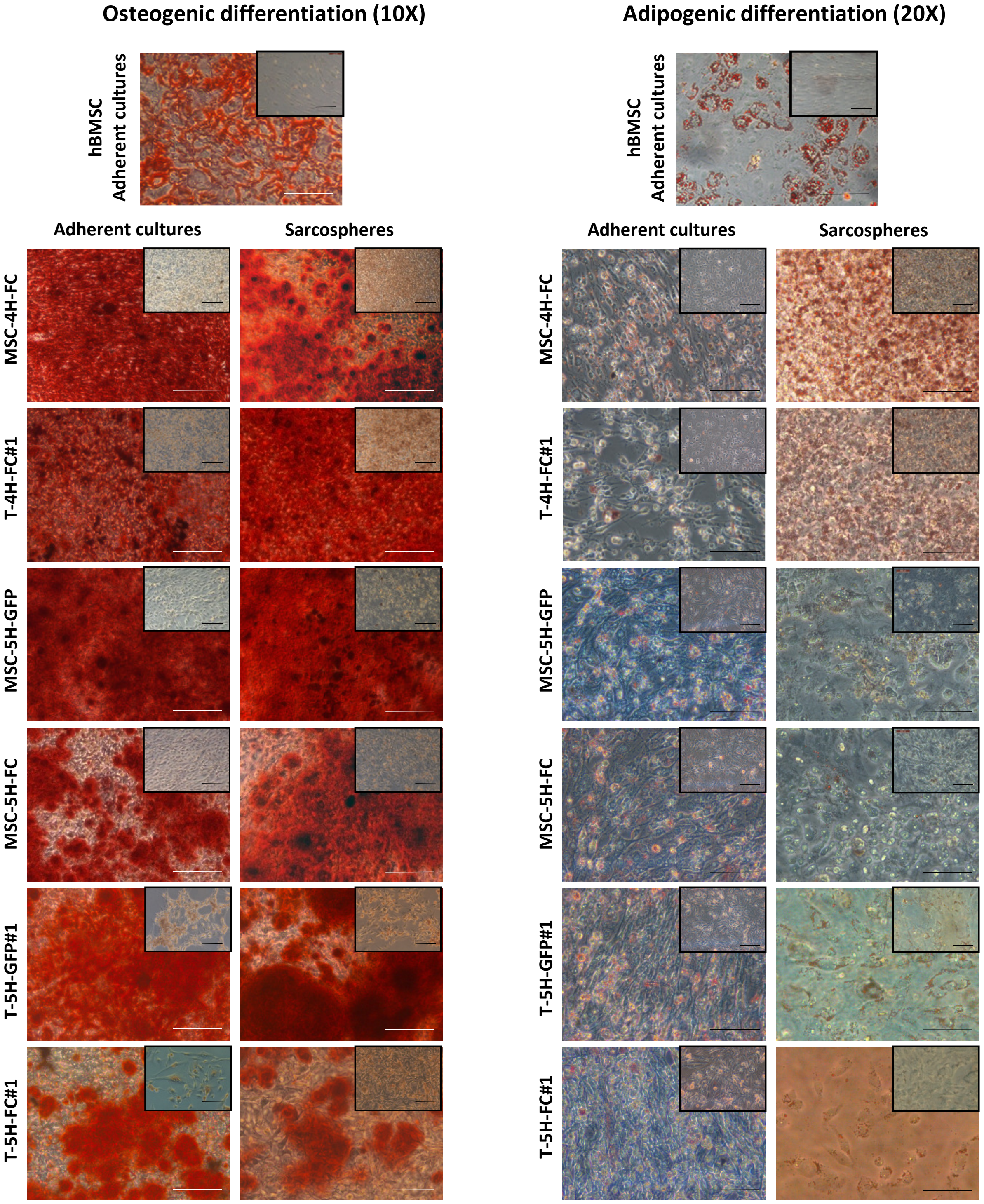

Figure S4: Representative images of the osteogenic (alizarin red staining, left panels) and adipogenic (oil red staining, right panels) differentiation capacity of adherent and tumorsphere cultures of the indicated cell types. Adherent cultures of hBMSCs were used as positive controls of differentiation. Inset images represent negative controls of differentiation. Scale bars=100 µm for osteogenic differentiation and =50 µm for adipogenic differentiation.

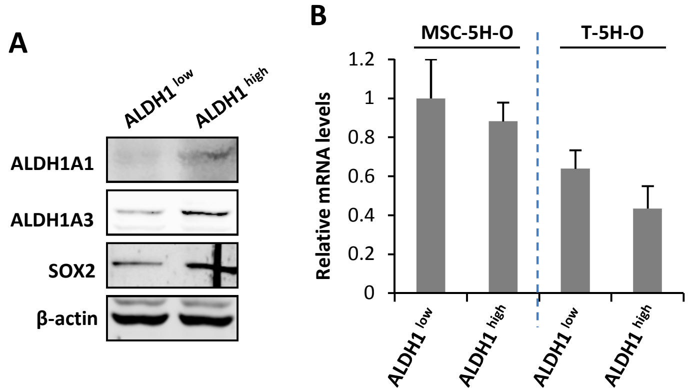

Figure S5. Expression of ALDH isoforms and pluripotency factors in ALDH^{low} and ALDH^{high} subpopulations. (A) Protein levels of the indicated factors in sorted populations of MSC-5H-O cells. β-actin levels were used as loading control. (B) Taqman-probe based qRT-PCR analysis of the expression of KLF4 in sorted populations of the indicated cell lines. mRNA levels were referred to MSC-5H-O ALDH^{low} levels. Error bars represents the standard deviation (n=2 independent experiments). MSC-5H-O cells displayed 2-fold more expression than T-5H-O cells. In any case, no significant variation was detected between the ALDH^{low} and ALDH^{high} subpopulations of any cell type.

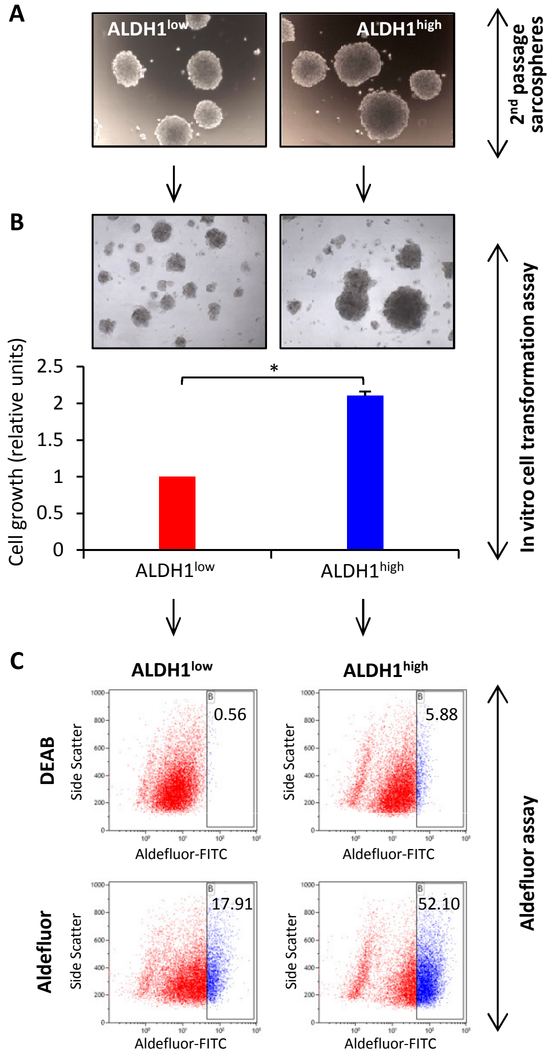

Figure S6: ALDH1^{high} cells displayed increased tumorogenic properties. T-5H-O cells were sorted by flow cytometry according to their ALDEFLUOR activity in ALDH1^{high} and ALDH1^{low} populations. Sorted cells were then growth as tumorspheres for two successive passages (A), disaggregated and assayed for soft agar colony formation (B) and finally, recovered from soft agar cultures and assayed for ALDEFLUOR activity (C). Error bars represents the standard deviation and asterisks indicate a statistically significant difference between the indicated conditions (*:$p<0.05$, **:$p<0.001$; two-sided Student t test).

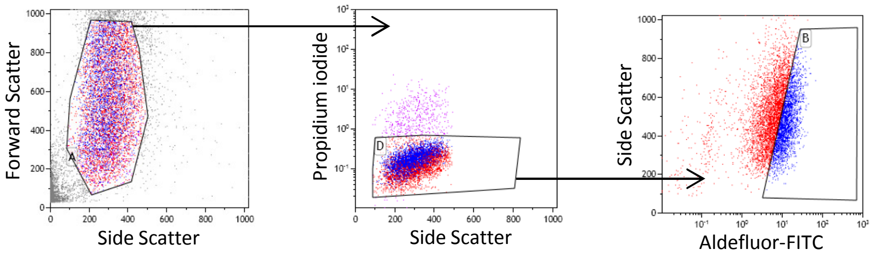

Figure S7: Analysis of ALDEFLUOR activity. Gating strategy used to analyzed ALDEFLUOR activity. Cells positive for propidum iodide (0.5 µg/ml) staining were excluded from the analysis.
